# Fiftieth Anniversary of the Oneida County Medical Society

**Published:** 1856-08

**Authors:** 


We cut from the *Utica Morning Herald*, the following interesting account of the semi-centennial anniversary of the Oneida County Medical Society.

That time-honored organization has ever numbered among its members some of the noblest spirits in the profession, and has always stood in the front rank in point of talent, and especially in enforcing professional discipline. To this it owes its health and vigor.

*Fiftieth Anniversary of the Oneida County Medical Society.*—The annual meeting of the Oneida County Medical Society, was held yesterday, at Bagg's Hotel. Dr. Cobb, the retiring President, delivered a brief and appropriate address, and the following officers for the ensuing year were chosen:

President—N. H. Dering.
Vice-President—James S. Whaley.
Treasurer—D. G. Thomas.
Librarian—S. G. Wolcott.
Drs. McCall, Coventry, Burke, Porter, and C. M. Barrows, Censors.

*Organization.*—Dr. D. G. Thomas, chairman of the committee of arrangements, in behalf of the committee, welcomed the members of the society, not as strangers but as brethren, fellow-laborers in the same calling, members of the same noble profession, to commemorate an important professional movement, to do honor to the founders of this society, and to greet their survivors. Fifty years, the 6th inst., have passed since this society was organized; and arrangements have been made to celebrate the semi-centennial celebration. Dr. T. concluded by nominating as President for the Festival, Dr. Arba Blair, of Rome, who was conducted to the chair by Drs. McCall and Coventry. Dr. Blair, on taking the chair, alluded to the fact that himself and Dr. Alexander Whaley were the only members of the society then present, who took part in its organization.

Drs. Alex. Whaley, Samuel Beach, Thomas Goodsell and Seth Hastings, were chosen Vice-Presidents, and M. M. Bagg, Secretary.

Letters were read in response to invitations to attend this anniversary, from Drs. Laurens, Hull, and H. Norton, and from Hons. P. Gridley, J. W. Williams, S. Beardsley, and H. Denio.

After a brief recess, Dr. D. G. Thomas delivered the anniversary address. After glancing at the events of the past half century, as bearing upon the profession and its practitioners, he spoke of the great dignity of the medical profession, and sketched the rise of medical societies in this state. He then epitomized the laws bearing upon the medical profession as such. Many passages of the address, on the scope and importance of the medical profession, were very happy. As the whole address is to be published, we yield the more readily to the necessity which compels us to omit a lengthy notice of it.

On motion of Dr. Dering, a vote of thanks was extended to Dr. Thomas for his interesting and eloquent discourse, and a copy was requested for publication. Drs. Dering, Coventry and Bagg, were appointed a committee on its publication.

*The Dinner.*—At this point the society sat down to a sumptuous dinner, including the delicacies and substantials of the season, and quite worthy of the reputation of Bagg's Hotel. Among the invited guests were Mayor Hubbell, C. H. Doolittle, Esq., and several representatives of the press. Grace was said by Rev. Mr. Barrows.

After dinner had been discussed, the regular toasts were read by Dr. Thomas, and responses were made as follows:

TOASTS AND RESPONSES.

1. The First Meeting and organization of the Oneida County Medical Society.

Dr. Blair responded. He found himself almost the only person present to-day who took part in the organization of the society. He glanced at the early meetings of the society, and the moral and personal worth of the originators; but he thought the society had much improved, and the profession had risen in the public estimation. He spoke of the benefits conferred by the County and State Medical Society, particularly in years gone by, when text-books were scanty and scarce. He spoke feelingly of the progress of the profession, and of the present advantages it offers to its practitioners.

2. The Survivors of the First Meeting of the Society.

Dr. Sturdevant, of Rome, spoke of the origin of the Medical Society, of the prosecution of the work, and of its results. Only four links remain to bind the society to its first meeting. The society now contains many men of the highest acquirements. To the originators of this society and others pursuing the same course, we are indebted to the high position of the medical profession in this country. Its schools are numerous and excellent, and its literature equal to that of any other country. Dr. S. paid a handsome tribute to the fathers of the society.

3. The Memory of our Co-Laborers who have gone to their reward.

Drank standing.

4. The Medical Profession; Past, Present, and Future.

Dr. Goodsell said the past history of medical science in Oneida county, stood second to none in the eyes of scientific men throughout the state and country. And he had frequently received compliments to the faculty of this county when abroad. For the present, he thought, if the profession here do lack the warm and kindly feeling proper in counsel and in intercourse, the fault should be corrected. Quackery, he thought, was now in high favor, from its great promises and the ignorance of the masses. The clergy and the lawyers have to some extent aided in this result. He regretted those professions treated medicine as a trade, and not as a science.

For the future, Dr. G. was not over confident. He was apprehensive there are too many medical colleges. For the sake of numerical show, they are granting their diplomas too cheaply. He recommended young men to go to the schools where is found the highest talent. He did not see how the educated man would fare with the quack, while the people are as now ignorant. He must do right in the light of science, and take the consequences.

5. Medical Education.

Dr. Coventry spoke very handsomely of this extensive subject; considering what is Medicine? Medical Education? Where can this Education be obtained? He showed that medicine is a learned profession; the classical languages are necessary to its study; the natural sciences are requisite to it; it is a continual application of the principles of inductive philosophy. There are two classes of medical men, the discoverers of principles and remedies; and secondly, those who simply apply them. The true physician is a scientific man; the empiric is a mere medicine man, wandering after some arcana never to be discovered, and always neglectful of scientific principles. Dr. C.

said the age of witchcraft may be said to have passed away; but human nature is very much the same; and the magical or mysterious class of doctors still remain. He followed in a thorough analysis of the three classes of physicians, the magical, the empirical and the philosophical. He concluded by pointing to thoroughly educated physicians, and medical colleges, as the place for obtaining proper medical education.

6. The State Medical Society.

Dr. McCall returned thanks to the senior body here assembled, for the honor expressed to the State Society in this sentiment. He stated the character of the State Society. He spoke of the advance of science, as the result of the positive philosophy.

7. The Public Press.

John B. Miller responded, closing with the sentiment:

The Physical Sciences and those who practice the Art of Physic: The Press will always cheerfully chronicle the progress of the one and the triumphs of the latter.

8. The American Medical Association.

Dr. Dering responded in an elaborate and elegant notice of the National Association, its meetings and its advantages.

9. Invited Guests.

C. H. Doolittle in response, paid a tribute to the society and to the medical profession, and spoke of the reflex obligations of the community to the scientific physician. He closed by giving this sentiment:

The Medical Profession: Its benevolent actions, its arduous duties and weighty responsibilities, its relation to man's temporal condition and the broad field of scientific investigation it embraces, not only secures to it the respect and confidence of mankind, but should secure it a remuneration commensurate with its dignity, intelligence and exacting duties, and should give it that protection from empiricism which an intelligent people can give.

10. Medical Hygiene.

Dr. Thomas said a few words in connection with this sentiment.

11. The Responsibility of Medical Men.

Dr. Gardner responded to this sentiment, dwelling on the responsibility of the medical man to be thoroughly educated in all science, and to have high motives in the practice of his profession. Dr. G. was earnest, forcible and effective in his remarks.

12. The Clergy.

The gentleman expected to respond to this sentiment not being present, Dr. Coventry spoke tastefully and forcibly of the relations between the clerical and medical professions.

13. The Ladies.

Dr. McCall was gallant enough to respond, paying a deserved compliment to our healthy and sensible mothers and grandmothers.

Dr. Coventry proposed:

Our distinguished guest: The Mayor of the city of Utica.

Mayor Hubbell said he was no speech-maker; but expressed the satisfaction he had felt in the exercises of the occasion. He told the story of Judge Staring's toast, which he dipped out with his hands; and referring to the cholera season in this city, paid a handsome compliment to the self-sacrificing devotion of our physicians. They deserve to stand high in the community, and to be better remunerated than they are. The Christian physician has besides the consciousness that he will have a reward hereafter. He gave as a sentiment:

The Family Physician: The most confidential relation that can exist in society.

Dr. Bissell offered the following:

Legitimate Medicine: The True Application of Science to the Art of Healing.

Col. Jewett sent the following:

The Doctor: God bless him. When armed with science, skill and kindness, he is ever a ministering angel to the afflicted.

Dr. McCall read a sketch of Medical Character.

Dr. Maltbie read an address, concluding with:

The Old Medical Gentlemen of Oneida County, who have grown grey in relieving human agony and promoting human health and happiness.

And then the celebration closed.

